# The legacy from the 50 years of acid rain research, forming present and future research and monitoring of ecosystem impact: *This article belongs to Ambio’s 50th Anniversary Collection. Theme: Acidification*

**DOI:** 10.1007/s13280-020-01408-7

**Published:** 2020-12-08

**Authors:** Bjørn Olav Rosseland

**Affiliations:** Faculty of Environmental Sciences and Natural Resource Management (MINA), Norwegian University of Life Sciences (NMBU), Sørhellinga, Postboks 5003 NMBU, 1432 Ås, Norway

**Keywords:** Acidification, Biology, Effects, Freshwater, Monitoring, Recovery

## Abstract

Acid rain and acidification research are indeed a multidisciplinary field. This field evolved from the first attempts to mitigate acid freshwater in the 1920s, then linking acid rain to the acidification in late 1950s, to the broad project-concepts on cause and effect from the late 1960s. Three papers from 1974, 1976 and 1988 demonstrate a broad approach and comprise scientific areas from analytical chemistry, biochemistry, limnology, ecology, physiology and genetics. Few, if any, environmental problems have led to a public awareness, political decisions and binding limitations as the story of acid rain. Acid precipitation and acidification problems still exist, but at a lower pressure, and liming has been reduced accordingly. However, the biological responses in the process of recovery are slow and delayed. The need for basic science, multidisciplinary studies, long time series of high-quality data, is a legacy from the acid rain era, and must form the platform for all future environmental projects.

Three papers in *Ambio*, Almer et al. (1974), Schofield (1976) and Henriksen et al. (1988), represent a very important “state of the art” at their time, focusing on the huge environmental effect caused by long range transported air pollutants, called “acid rain”. In Scandinavia, acid water had been recognised as a problem for especially Atlantic salmon (*Salmo salar*) and brown trout (*Salmo trutta*) since the 1920s (Dahl 1927), and hatcheries had locally installed limestone-filters to improve hatching success. Researchers had first speculated that reduced fish populations were caused by fish diseases or over-fishing, until Dannevig (1959) claimed the acid precipitation to be a major factor in causing the problems. However, the focus set by Professor Svante Odén in Sweden in 1968 was unique, leading to extensive investigations, documentation of status and onset of national projects in Sweden, and later in Norway (Grennfelt et al. 2020). The Almer et al. (1974) paper contained high-quality chemical data from Swedish lakes, sampled in a systematic way and linked to biological data from many of the same lakes, including primary producers (algae), invertebrates and fish. Observations of increased clarity of lakes formed the hypothesis of “oligotrophication”. Increased concentrations of several trace elements like Pb and Zn were also described. This paper was extremely important for the onset of similar projects and programs in many countries suffering from acid rain. It raised the international awareness to the top political level, leading finally to the international agreements on reductions in sulphur (S), nitrogen (N), ozone, heavy metals and Persistent Organic Pollutants (POPs).

The study of a major fishkill of brown trout in the Norwegian River Tovdal during snowmelt in 1975, enabled a first understanding of the physiological responses to these episodic events of low pH and ionic dilution (Leivestad and Muniz 1976). This article was central in the review paper in *Ambio* by Schofield (1976), where he pointed to the common phenomenon of increasing numbers of barren lakes in Scandinavia, as in the eastern North America. He also concluded that the youngest life stages, egg to larvae, were the most sensitive, leading to reproduction failure and lacking year-classes. Species and strain differences in sensitivity to acid water were discussed, but because of the rapid acidification of the environment, natural selection of more tolerant strains or populations seemed not to have occurred. The reference list reflects a huge ongoing research into the field, as many papers were termed “in press” or “in preparation”.

Indeed, this was the start of an era where field data and laboratory studies were paralleled. Until 1977, the explanation for the negative biological effects of acid water was the low pH combined with low conductivity and calcium. A breakthrough came in 1977, when a report from Schofield (1977) suggested that aluminium (Al) could be THE key factor for the toxicity in acid water. Overnight, physiological groups in many countries included Al in their experiments. Another breaking news was the discovery of the importance of the chemical speciation of the Al, separating organic bound Al, from the non-organic, termed labile Al (LAl) or inorganic Al (Ali), and that the freshwater toxicity of Al was linked to LAl (Driscoll et al. 1980). New laboratory methods were developed to identify these species (e.g. Driscoll 1984).

Liming of acid waters started as a test program (1976–1981) in Sweden (Henrikson and Brodin 1995). Large lakes with a retention time of several years were their first target. When the Norwegian liming project started, 1978–1984, the cooperation with Sweden was strong, and has been like that ever since. The small sized lakes with heavy rainfall and retention times in months in Norway, called for other criteria and methods, including liming of large rivers (Baalsrud et al. 1985). The Swedish and Norwegian mitigation programs have been a great success and became the fundament for liming programs in both the USA and Canada.

In the early 1980s, there was a mass mortality of Atlantic salmon in River Vikedal (Norway) in their smolt-stage, prepared to migrate into seawater. This changed the focus from eggs and juveniles, to smolts being the most sensitive life history stage (Rosseland and Skogheim 1984). This meant that the water quality in rivers housing Atlantic salmon had to be protective for smolts from early spring until June, the period where smoltification and migration occurs. Eight years later, the phenomenon of “Mixing Zones” was described (Rosseland et al. 1992). A chemical inequilibrium zone formed downstream the mixture of an acid stream and a neutral or limed river resulted in Al polymerization and an initially extreme toxicity. Such zones could kill year-classes of smolts while migrating during spring floods. Liming all side tributaries to avoid such zones increased the cost of liming rivers.

The problems associated with acidification called for long-term monitoring, and national programs started in the beginning of the 1980s. Lakes and rivers, not influenced by liming, formed the basis for annual or periodic sampling of water and biota. ICP Waters, “the International Cooperative Program for Assessment and Monitoring of the Effects of Air Pollution on Rivers and Lakes”, started in 1984 and included water chemistry and biota.

The Norwegian 1000-lake study by Henriksen et al. (1988) was launched at a time when large databases from Norwegian lakes existed (i.e. Wright and Snekvik 1977), and where the scientific understanding of the relations between chemical elements and their biological effects was greatly improved, relative to the 1970s, and where politicians had agreed and started to reduce emissions. The study of Henriksen et al. (1988) included up-to-date chemistry data and a lake selection, similar to that in Almer et al. (1974) paper. Unlike lakes in Sweden and northeastern America, the Norwegian lakes had low TOC (Total Organic Carbon) and low conductivity waters with few species of fish, mainly brown trout and Arctic charr (*Salvelinus alpinus*). The lakes selected included reference lakes and covered the whole country. Besides water sampling in autumn, great effort was put into information of fish status from the same lakes, which resulted in a second paper in *Ambio* one year later (Henriksen et al. 1989). These two papers, where chemical data were directly linked to fish status, gave a basis for the application of a series of models like MAGIC (Model of Acidification of Groundwater in Catchments, Cosby et al. 1985) for the prediction of past and future environmental changes caused by acid rain. The monitoring program in Norway chose 100 lakes from these studies, to be followed at regular sampling intervals. Problems occurred, however, as some lakes became influenced by upstream liming or other catchment changes. True references over time are difficult to maintain.

All three papers (Almer et al 1974; Schofield 1976; Henriksen et al. 1988) reflect an environmental status from a time of high S and N deposition, in catchments still in the process of acidification. In the 1970s, before the international acceptance and agreement on reductions in emissions, we were not fully aware of the large time lag between change in precipitation chemistry, catchment reactions, water chemistry change and biological responses. Results from test fishing had revealed reproduction failure nearly 20 years previously to sampling (Rosseland et al. 1980). This illustrates that fish status cannot be reliably predicted from present lake chemistry. Prognoses of biological recovery, as depositions became reduced, had therefore to be corrected, as the recovery followed a pattern of hysteresis. Models for restoring a population of Atlantic salmon, ASRAM (“Atlantic Salmon Regional Acidification Model») Korman et al. (1994) forecasted 12–15 years of recovery, given no episodes of critical water quality for the most sensitive life stage. One critical episode could, however, delay Atlantic salmon recovery by another 10–15 years, a recovery delay also found for brown trout by the FIB-model (Raddum and Rosseland 2005). Recovery of fish was consistently associated with increasing pH, reduced Al, increased TOC (Keller et al. 2019), but also a decreased Ca concentration (Jeziorski et al. 2008). As Ca is a physiological important protective ion for aquatic organisms, this could slow down the biological recovery, partly explaining why fish and invertebrate populations struggled, despite “good” and improved water quality. Today’s practice with downscaling of liming in accordance with the reduced emissions will therefore depend on careful monitoring of the biological communities and their most sensitive species and life history stages for evaluation if end of liming was correct (Anderson et al. 2002).

More developed genetic and physiological methods and chemical speciation tools have enabled a deeper understanding of species-, strain- and life history stage sensitivities. The extreme sensitivity towards Al for Atlantic salmon smolts prior to sea migration (Kroglund et al. 2008) is caused by the formation of the “supersensitive seawater isoform” of Na–K–ATPase (Nilsen et al. 2010), the major ion-regulatory enzymes in gills. Experiences from Al toxicity studies are now a model for testing of other metals, radioactive substances and organic substances in fresh or marine waters.

The scientific era of acidification, starting in the early 1970s and represented by the three papers I reflect on here, forms the basis of many environmental programs until today. Long time series of data (chemistry or biota) based on international protocols, and permanent sites with minimum disturbances, are mandatory for any modelling and forecasting of environmental or climate change. Huge international research programs and manipulation studies, many partly financed by the EU, have studied catchment responses to increased or decreased occurrence of, e.g. S, N, TOC and CO_2_, as well as changing temperature. The development of techniques of detoxifying acid water through lime, silica lye, use of seawater, etc., have been an extremely important factor for the success of Atlantic salmon smolt production worldwide. In 2017, 15% of the wild Atlantic salmon caught by anglers in Norway was in limed rivers.

Data from one of the longest running chemical and biological data series (Lake Saudlandsvatn, Hesthagen et al. 2011, Fig. 1) were used to illustrate positive changes in European environments (Maas and Grennfelt 2016) and demonstrated a process of recovery from acidification in biota. However, the figure does not show the lack of older age classes in brown trout, dying after spawning, as a result of still marginal chemical conditions for this sensitive life history stage.

**Fig. 1:**
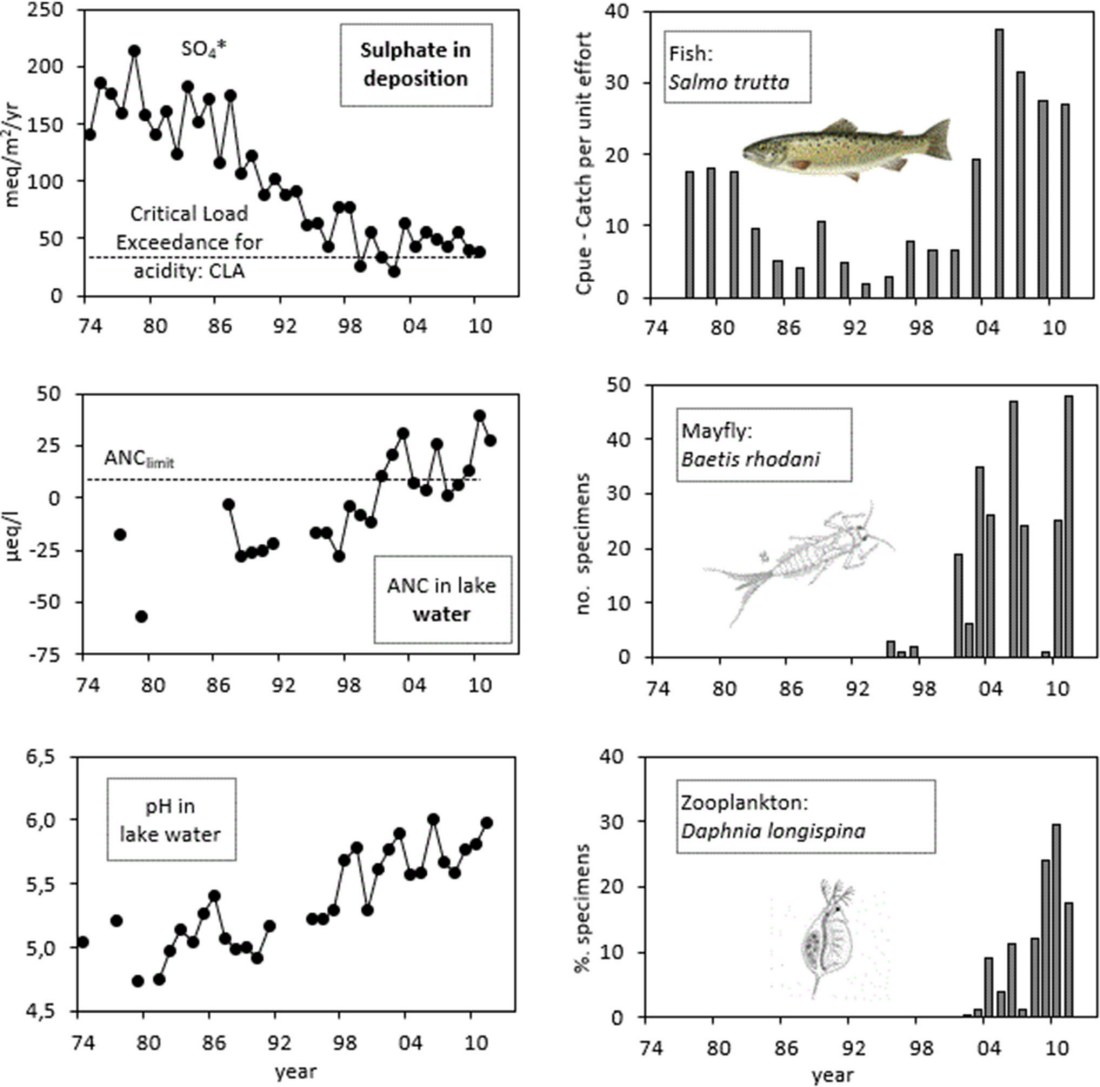
This figure, modified from Hesthagen et al. (2011), was used in the report by Maas and Grennfelt (2016) to illustrate recovery from acidification at Lake Saudlandsvatn, Southern Norway. As sulphur deposition has decreased, so the acid neutralising capacity (ANC) and pH of the lake water have increased, and the populations of the three sensitive species have begun the process of recovery. The figure looks very promising, also for brown trout, but the increase in catch from 1994 and onwards was mainly by young fish. Lacking adult post-spawners indicates marginal chemical conditions to a sensitive life history stage

In 2019, a “new” 1000-lake study was performed in Norway. The legacy from the acidification research will forever be a “lesson to be learnt” for future environmental science programs, and reference for modelling of our future environment.
